# Willow Bark for Sustainable Energy Storage Systems

**DOI:** 10.3390/ma13041016

**Published:** 2020-02-24

**Authors:** Mathias Andreas Hobisch, Josphat Phiri, Jinze Dou, Patrick Gane, Tapani Vuorinen, Wolfgang Bauer, Christian Prehal, Thaddeus Maloney, Stefan Spirk

**Affiliations:** 1 Institute of Paper, Pulp and Fibre Technology, Graz University of Technology, Inffeldgasse 23, 8010 Graz, Austria; mathias.hobisch@tugraz.at (M.A.H.); wolfgang.bauer@tugraz.at (W.B.); 2 Department of Bioproducts and Biosystems, Aalto University, Vuorimiehentie 1, 02150 Espoo, Finland; josphat.phiri@aalto.fi (J.P.); jinze.dou@aalto.fi (J.D.); patrick.gane@aalto.fi (P.G.); tapani.vuorinen@aalto.fi (T.V.); 3 Institute for Chemistry and Technology of Materials, Graz University of Technology, Stremayrgasse 9, 8010 Graz, Austria; christian.prehal@tugraz.at

**Keywords:** willow bark, upcycling, carbon activation, electrode formation, supercapacitors, organic electrolytes

## Abstract

Willow bark is a byproduct from forestry and is obtained at an industrial scale. We upcycled this byproduct in a two-step procedure into sustainable electrode materials for symmetrical supercapacitors using organic electrolytes. The procedure employed precarbonization followed by carbonization using different types of KOH activation protocols. The obtained electrode materials had a hierarchically organized pore structure and featured a high specific surface area (>2500 m^2^ g^−1^) and pore volume (up to 1.48 cm^3^ g^−1^). The assembled supercapacitors exhibited capacitances up to 147 F g^−1^ in organic electrolytes concomitant with excellent cycling performance over 10,000 cycles at 0.6 A g^−1^ using coin cells. The best materials exhibited a capacity retention of 75% when changing scan rates from 2 to 100 mV s^−1^.

## 1. Introduction

The forestry industry is one of the most prominent worldwide operating sectors with annual turnovers in the range of tens of billions of euros. While there are many areas where wood is commercially used in large-scale industries (e.g. furniture, construction, pulp and paper, etc.), some of the waste and byproducts do not have any major application at present. One of these remaining materials is bark, which is mostly used for incineration. Therefore, research has focused on using bark to produce chemicals like acetic acid [1] or biobased oils [2]. Others investigated the fire resistance of bark in dependence of the water content and the thickness of the bark [3,4]. This was also implemented in developing a fire-resistant composite on the basis of suberin and lignin for wooden construction materials [5]. An overview of existing bark applications was recently provided by Pásztory et al. [6].

The development of energy storage and conversion technologies is a key challenge to address climate change and its associated negative impact on the environment, society and economy.

Electrical double-layer capacitors (EDLCs), are a major storage technology and have a different working principle than batteries, as the energy is purely physically stored in an electrical double layer at the carbon–electrolyte interface. As charging and discharging do not cause any stress on the bulk material, these supercapacitors (SCs) have much higher cycling stability than batteries. The absence of slow intercalation-type chemical reactions also allows for faster charge/discharge compared to batteries, resulting in large power densities. In principle, the physical storage mechanism makes SCs less sensitive to temperature extremes, particularly compared to lithium-ion batteries. A disadvantage of the capacitive storage mechanism in SCs is the relatively low energy density compared to batteries. Efficient energy management relies on the complementary use of batteries and supercapacitors in the future to cover the whole range of energy densities, power densities and charging/discharging times [7,8,9].

Many SC systems employ aqueous electrolytes such as H_2_SO_4_ and KOH. These are cheap and available, but have two intrinsic disadvantages: they are corrosive and the operating voltage is limited to the decomposition of water (1.2 V). In contrast to commercial SC materials, biomass-derived carbons have not been fully investigated with organic electrolytes. Organic electrolytes are not corrosive to the casing and have a larger operating voltage than aqueous electrolytes. SCs mostly employ activated carbon electrodes with high specific surface areas and a large fraction of micropores (pores < 2 nm) to achieve highest capacitances. Ideally, these carbons also feature a hierarchical pore size distribution to allow for optimum ion transport and SC rate performance. As a source of the activated carbons, biomass precursors are often used. Examples include specialties (e.g., nanocellulose) and mass products (e.g., paper, textile fibers), as well as waste materials from biorefineries or agriculture [10,11,12]. A comprehensive review covering the literature on the use of polysaccharides as source for these materials showed that there is still large potential for valorizing waste materials into high-performance electrode materials [13].

Bark and its extracts have also been tested for supercapacitor assembly, using the hierarchical structure of bark for carbonization and activation [14,15,16,17]. Several activating agents (KOH, H_2_SO_4_, NaOH, H_3_PO_4_ and ZnCl_2_) have been applied so far for the activation of biowaste materials [18], with the main focus now being placed on KOH, which has been proven to be the most promising one for bark [19]. The accessibility of nanopores for the electrolyte is of utmost importance. A 1:2 ratio of bark:KOH revealed the highest capacitances during the assembly of aqueous supercapacitors [15,17].

In the present work, we chose willow as a typical representative of western wood, with more than 500 species distributed all over the world. Its high abundance makes it one of the most processed wood species in the world for the pulping process, which allows it to be harvested directly after debarking without any further purification steps. Some of us have already developed a procedure to obtain high-surface-area activated carbons from non-purified willow wood for use in aqueous electrolyte supercapacitors [20]. The performance was excellent for aqueous-based systems but has intrinsic limitations in terms of energy density and operating voltage. The scope of this work is to extend the knowledge from earlier studies and investigate the potential of willow bark as an electrode material for supercapacitors using organic electrolytes.

## 2. Materials and Methods

### 2.1. Materials

The willow bark (WB) used in this study was a one-year-old willow hybrid “Klara”, harvested from the plantation of Carbons Finland Oy. HCl and KOH were purchased from VWR (Radnor, PA, USA). (ARKEMA, Colombes, France) and Super P carbon black (Timcal, Bodio, Switzerland) were used for the electrode formation. Acetonitrile (ACN, 99.9%, Sigma-Aldrich, St. Louis, MO, USA), tetraethylammonium tetrafluoroborate salt (TEABF_4_, 99%, Alfa Aesar, Haverhill, MA, USA) and glass microfiber filters (separator, from Whatman, Maidstone, UK) were used for battery assembly.

### 2.2. Preparation

**Figure 1:**
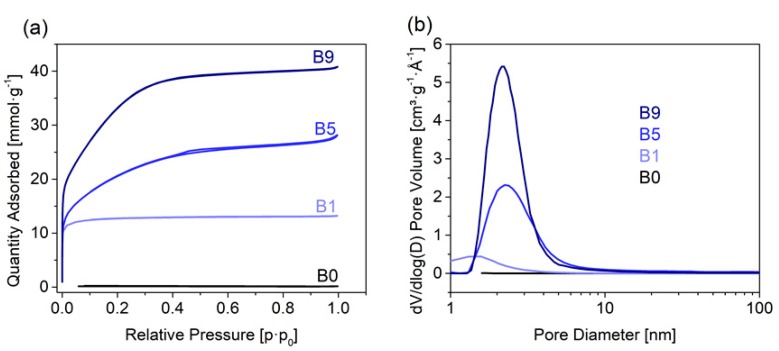
(**a**) Nitrogen physisorption isotherms and (**b**) corresponding pore size distribution for the willow bark and all three activated samples.

The willow was debarked immediately after harvesting. The dried willow bark was ground into powder using a Wiley mill, to an average particle size of 500 µm. Carbonization was accomplished using a two-step method. The schematic preparation of the willow-bark-derived activated carbon is shown in Figure 1. In the first step, the WB was pre-carbonized at 600 °C for 1 h at a heating rate of 5 °C min^−1^ under nitrogen flow, followed by a thorough washing with water and drying in the oven at 105 °C. In the second step for activation, KOH was dissolved in about 20 mL of deionized water and thoroughly mixed with the dried pre-carbonized carbon in various KOH/C mass ratios of 0, 1, 5 and 9. The samples were then activated at 800 °C for 1 h at a heating rate of 5 °C min^−1^ under nitrogen flow and allowed to cool down to room temperature naturally. After carbonization, the samples were thoroughly washed with 1 M HCl and subsequently with deionized H_2_O until neutral pH was reached. The powders were then dried at 105 °C for at least 24 h. The resulting activated carbon samples are designated as B0, B1, B5 and B9 with the numeration corresponding to the KOH/C ratios.

### 2.3. Electrode Preparation

The samples were hand milled, 10 wt. % PTFE and 10 wt. % Super P were added and a dough was formed in isopropanol. This dough was rolled out and dried overnight. Electrodes were stamped, weighed and vacuum-dried with an average loading of 4 mg cm^−2^.

### 2.4. Material Characterization

#### 2.4.1. Evaluation of Accessible Surface Area

Pore volume and surface area were determined by nitrogen gas adsorption at 77 K using a Micromeritics Tristar II apparatus (Micromeritics, Unterschleißheim, Germany). Specific surface areas were obtained using the BET method. BET areas typically overestimate absolute values of the specific surface area in microporous carbons, but reliably characterize their relative order. Pore size distributions of the investigated carbons were again generated by the BJH method [21]. X-ray photoelectron spectroscopy (XPS) was conducted using a Kratos Axis Ultra ESCA system (Kratos, Manchester, UK) with a monochromatic Al Kα source.

#### 2.4.2. Scanning Electron Microscopy

The scanning electron microscopy images were analyzed with a Zeiss Sigma VP (Zeiss, Jena, Germany) at 5 kV acceleration voltage using a SE2 detector. The samples were sputtered with platinum to form a conducting film prior to SEM measurements.

#### 2.4.3. Cell Preparation

The cells were assembled using a Swagelok-type three-electrode test cell (Swagelok, Solon, OH, USA) with partially delithiated Li_1-x_FePO_4_ as the reference electrode and two symmetrical activated carbon electrodes as counter and working electrode with a separator in-between, and soaked with 80 µL electrolyte solution (1 M TEABF_4_ in ACN). The coin cell consisted of the two symmetrical activated carbon electrodes and the same separator; everything was soaked with 80 µL of electrolyte solution. All cells were assembled in the glove box, avoiding moisture and oxygen.

All electrochemical cells were characterized by a Biologic MPG-2 potentiostat (BioLogic, Seyssinet-Pariset, France) at ambient temperature. Cyclic voltammetry and galvanostatic cycling experiments were conducted at ambient temperature in the Swagelok-type 3-electrode. Cyclic voltammetry (CV) as well as galvanostatic charging–discharging (GCD) measurements were performed between 0 and 2.3 V. The scan rate varied from 2 to 100 mV s^−1^ and the current density from ~0.1 to 6 A g^−1^ per mass of active material in the cell. Long-term cycling was carried out in a coin cell at 0.6 A g^−1^ of the active material in the cell over 10,000 cycles between 0 and 1.4 V. Cyclic voltammetry was used to determine the capacitance according to Equation (1):

(1) $$C_{S,\mathrm{Electrode}}\;=\;2\cdot \;\frac{\int_{V_{i}}^{V_{f}}i\;\cdot \;dV}{\Delta V\;\cdot \;v\;\cdot \;m}.$$

The specific capacitance C_s_ of one electrode was determined by the integral of the CV curve, limited by *V_f_* and *V_i_* of the cell voltage Δ*V*, scan rate *v* and the mass of the active material *m* in the cell.

The supercapacitors’ rate-handling capability was tested by galvanostatic cycling at current densities ranging from 0.3 to 4 A g^–1^. The specific capacitance (F g^−1^) was calculated via Equation (2) with a shift in the current density ascribed to variations in the loading [22].

(2) $$C_{S,\mathrm{Electrode}}\;=\;4\cdot \frac{I\cdot \Delta t}{\Delta V\cdot m}$$

where *I* is the applied current, Δt the time for full discharge, ΔV the cell voltage and m the total mass of both electrodes.

## 3. Results and Discussion

### 3.1. Activation and Material Characterization

The dried and milled willow bark chips were converted into activated carbons at 800 °C. We used KOH as the activation agent, which was added in different ratios (1:0, 1:1, 1:5, 1:9, wt. bark: wt. KOH, samples denoted as B0, B1, B5, B9 in the following) to the milled bark. Specific surface area and pore volume increased with larger amounts of KOH used for activation, as previously reported. At the lowest KOH concentrations (B1), we obtained specific surface areas of 933 m^2^ g^−1^, which increased to 1584 m^2^ g^−1^ (B5) and 2564 m^2^ g^−1^ for B9. This sample also featured the highest pore volume with 1.48 cm^3^ g^−1^ (Figure 1).

**Figure 2:**
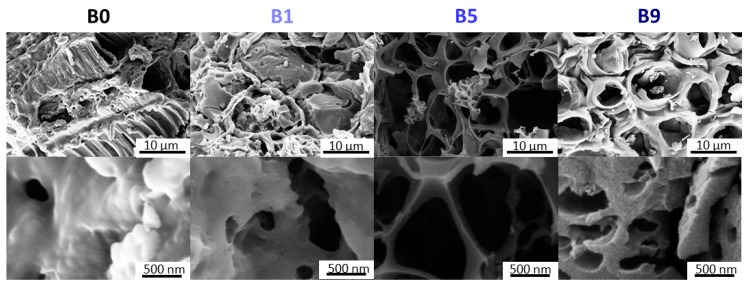
Scanning electron microscopy images of B0, B1, B5 and B9.

The morphology of the samples investigated via SEM reflected the results from BET, observing porous structures for all samples (B0–B9). However, a distinct transition from less-altered structures (B0, B1) to honeycomb-like structures was observed as soon as the bark:KOH ratio reached 1:5 (Figure 2—B5, B9). The porosity of the samples increased with the KOH concentration, showing the largest pores for B5. This structure was also retained for B9, but the pore size decreased. Consequently, B9 revealed the biggest surface area of all samples. At higher magnification, the pore system could be evaluated in more detail. The pores were larger in the case of B5 (several hundreds of nanometers) than for B9, where a hierarchical structure could also be observed.

**Figure 3:**
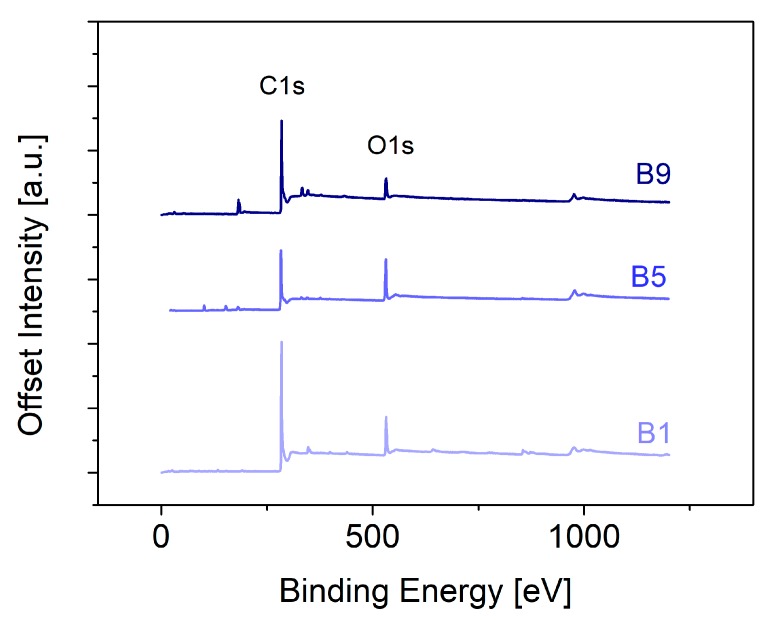
X-ray photoelectron spectroscopy data of the carbonized samples (B1, B5, B9).

The XPS data gave evidence of carbonization between the different samples (Figure 3). The carbonized barks featured carbon content from 70 at % (B1) to a nearly 90 at % C (B9), as obtained from evaluation of the C1s peak (283 eV). At the same time, oxygen levels were lowest for the variations in the oxygen content (O1s, 531 eV) and revealed the lowest amount for B9 (9 at %). Although the carbonization conditions were consistent, the composition was drastically different, indicating a great impact on the electrochemical properties of the materials.

### 3.2. Supercapacitor

**Figure 4:**
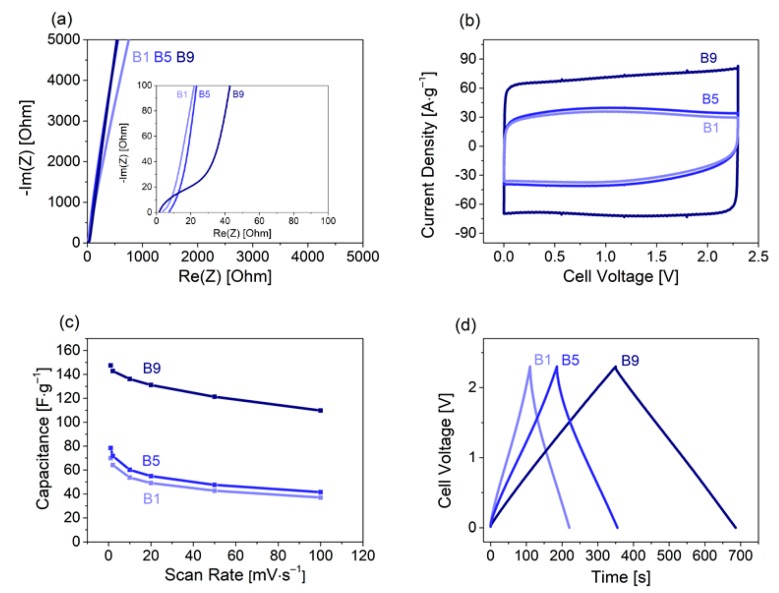
Electrochemical properties of all three manufactured activated willow bark samples, investigating (**a**) the impedance, (**b**) cyclic voltammetry (1 mV s^−1^), (**c**) associated capacitance retention and (**d**) a galvanostatic cycle at 1.5 mA.

The supercapacitors were investigated in a two- and three-electrode configuration using standard electrochemical methods such as impedance spectroscopy, cyclic voltammetry and galvanostatic charge/discharge. Electrochemical impedance spectroscopy revealed similarities for all materials with a slightly more distinct capacitive behavior for B9. A lower resistance and higher charge transfer resistivity (Figure 4a) was observed. The rectangular shape was typical for EDLCs and their capacitive energy storage (Figure 4b), and yielded 70, 72 and 147 F g^−1^ for B1, B5 and B9, respectively, at a cycling rate of 1 mV s^−1^. The lower current densities of B1 and B5 reflected the lower specific capacitances and indicated limitations for the use as supercapacitor electrodes. This was further illustrated by the decreasing current densities at higher cell voltages.

**Figure 5:**
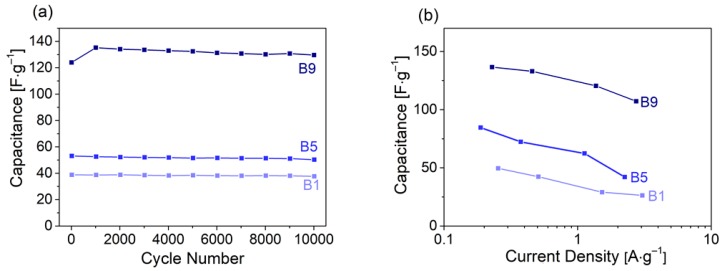
(**a**) Cycling behavior over 10,000 cycles, (**b**) the capacitance retention from galvanostatic cycling of willow bark samples activated with KOH.

The capacitance retention during a scan rate increase from 1 to 100 mV s^−1^ (Figure 4c) was lower for B1 and B5 (~54%) than for B9 (75%). Galvanostatic charge/discharge cycles demonstrated the characteristic triangular shape at 1.5 mA. This was associated with rising/falling potentials at turning points changing from charging to discharging and reverse (Figure 4d). The most significant characteristics for an SC electrode material are cyclability, rate handling capability and specific capacitance. For this purpose, the SCs were assembled in coin cells and cycled over 10,000 times at 0.6 A g^–1^. All materials showed high cycling stability (Figure 5a). B1 and B5 retained over 97% and 95% capacity, respectively, after 10,000 cycles. For B9, capacitance even increased about 8% over the first 1000 cycles, retaining 105% after 10,000 cycles. We speculate that surface reorientation during the first cycles led to enhanced accessibility of nanopores during cycling, thereby increasing capacitance. The hierarchical pore structure ensured short diffusion pathways into the bulk carbon material (SEM images, Figure 2), leading to reasonably good rate performances for all carbons (Figure 5b). The high specific capacitance of B9, caused by the large volume of micropores and the high specific surface area, was largely maintained, even at high specific currents.

### 3.3. Comparison with Other Biobased Supercapacitor Electrodes

Compared to other biobased materials in the literature, the reported activated willow bark in organic electrolytes performed excellently (Table 1). To the best of our knowledge, Wei et al. reported the highest capacitance for organic electrolytes with 236 F g^−1^ at 1 mV s^−1^ by a rather simple formation of hydrochar [23]. Electrode mass was not mentioned in that paper, although it may have had an impact on the performance of the supercapacitor [24]. Generally, higher electrode mass reduced the specific capacitance [10,11], while slower cycling rates increased the capacitance [25]. Based on the lower usable voltage window, aqueous electrolytes revealed generally lower specific energy than organic electrolytes. In the case of willow, the organic electrolyte (bark) outperformed the aqueous systems (wood) significantly (energy density, normalized by carbon mass—101 vs. 22 Wh kg^−1^ at a power density of 1000 W kg^−1^ for 1 M TEABF_4_/acetonitrile and 1 M aqueous Na_2_SO_4_, respectively) [20]. An overview is shown in Table 1.

## 4. Conclusions

We demonstrated that willow bark that was directly harvested at the manufacturing site could be used as starting material for the controlled synthesis of activated carbons. A prerequisite was the use of large amounts of KOH for activation to realize large surface area (>2500 m^2^ g^−1^) and a pore size distribution that was ideal for energy storage devices. Samples with such properties yielded supercapacitors with high capacitances (147 F g^−1^ at 1 mV s^−1^) and extraordinary capacitance retentions of 75% from 1 to 100 mV s^−1^. Galvanostatic cycling confirmed the excellent electrochemical properties. A large volume of micropores (and a large specific surface area) was responsible for the high specific capacitance. An ideal hierarchical pore structure permitted efficient ion transport and enabled the good rate handling capability of the material. Furthermore, capacitance retention was subsequently evaluated, obtaining an increase in capacitance of about 5% of the initial capacitance for the sample with the highest surface area (B9).

## Figures and Tables

**Table 1:** Overview of biomaterials tested as supercapacitor electrodes. ACN: acetonitrile, PC: propylene carbonate.

Carbon Source	Capacitance (F g^−1^)	Cycling Rate	Electrode Mass (mg cm^−2^)	Solvent	Reference
Cellulose, starch, eucalyptus wood	236	1 mV s^−1^	-	ACN	[23]
Herbaceous biomass waste	127	0.5 mA cm^−2^	11	ACN	[10]
Wood sawdust and tannic acid	140	0.2 A g^−1^	9–11	ACN	[11]
Birch wood sawdust	160	0.01 A g^−1^	4	ACN	[25]
Sucrose	120	1 A g^−1^	1.5–3	ACN	[12]
Leonardite fulvic acid	170	0.05 A g^−1^	-	PC	[26]
Carbon microfibers	172	1 A g^−1^	3	ACN	[27]
Willow bark	147	1 mV s^−1^	4	ACN	This work
Willow wood	394	1 A g^−1^	1.5–2	Aqu.	[20]
